# AnthroShift in a warming world

**DOI:** 10.1007/s44168-022-00011-8

**Published:** 2022-05-09

**Authors:** Dana R. Fisher

**Affiliations:** University of Maryland, College Park, Maryland, USA

**Keywords:** AnthroShift, Climate Action, Climate Politics, Activism, Climate-change adaptation

## Abstract

Thirty years after the UN Conference on Environment and Development created the UN Framework Convention on Climate Change, efforts to respond to the issue continue to be insufficient to meet the challenges of the climate crisis. This perspective builds on the experience of society’s responses to the COVID-19 pandemic to understand what is needed to get to meaningful climate action. It applies the framework of the AnthroShift to assess how transformational social change is likely to emerge. The paper concludes by determining that the most plausible pathway to an effective social response to the climate crisis would be driven by civil society. However, the level of mass mobilization needed is only possible if society is experiencing large-scale and sustained levels of risk that have tangible long-term consequences in terms of social cost to people and property.

## Introduction

In March 2020, the COVID-19 pandemic shut down the world. As populations around the globe hid inside their houses, the environmental benefits of society’s responses to this global pandemic were substantial (e.g., Bhat et al. 2021; Su et al. 2021; Venter et al. 2020). The threat of the new disease brought about a rapid societal transformation that included significant limitations on mobility and expansions of social welfare. At the same time, an unintended co-benefit of these measures to limit the spread of the disease was social change that put society on a much more sustainable trajectory in terms of its emissions of harmful greenhouse gases. As the majority of the global population experienced lockdowns and/or travel restrictions within and beyond their borders, energy consumption went down substantially (for a full discussion, see Sovacool et al. 2020; see Hosseini 2020 for an assessment of the ways the pandemic affected the transition to sustainable energy). Global, national, and local responses to the pandemic were in stark contrast to the slow and ineffective ways that the world has responded to the climate crisis. As youth climate activist Greta Thunberg shrewdly observed early in the pandemic: “The coronavirus is a terrible event....But it also shows one thing: That once we are in a crisis, we can act to do something quickly…we can act fast and change our habits and treat a crisis like a crisis” (as quoted in Goering 2020).

This paper uses lessons from society’s response to the COVID-19 pandemic to understand what it will take to get to meaningful climate action. It applies the framework of the AnthroShift to assess how transformational social change is most likely to emerge and considers what is the most reasonable pathway to an effective social response to the climate crisis. The paper is broken into three sections. First, I introduce the framework of the AnthroShift and apply it to the COVID-19 pandemic, paying particular attention to the environmental side effects. Second, I provide an overview of the social responses to the climate crisis and outline how the various social actors have responded thus far. Third, given the lack of sufficient climate action, I consider how the AnthroShift helps us think through what is needed to get to climate action that meets the scale of the crisis.

## The AnthroShift and COVID-19

Social responses to the pandemic and its environmental side effects are consistent with the notion of AnthroShift, which explains how social actors are reconfigured in the wake of widespread perceptions and experiences of risk (Fisher and Jorgenson 2019). When risk becomes so common that it is felt across society, the interrelations among the state, market, and civil society sectors shift substantially. The notion that risk can drive social change is consistent with the scholarly work on the Risk Society and Reflexive Modernization (e.g., Beck 1999; Beck et al. 1994; Beck 1997; see also Curran 2013; Elliott and Frickel 2015; Ekberg 2007; Rosa, McCright, and Renn 2015; For a full discussion regarding how the AnthroShift is related to Reflexive Modernization, see Fisher and Jorgenson 2019). Perhaps Ekberg best summarizes how this body of research interprets risk: it expands the “traditional concept of risk understood as the sum of the probability of an adverse event and the magnitude of the consequences, to include the subjective perception of risk, the inter-subjective communication of risk and the social experience of living in a risk environment” (Ekberg 2007, 343; see also Stallings 1990). It is worth noting that this viewpoint is consistent with much of the research on climate risk (see particularly Hultman, Hassenzahl, and Rayner 2010; for an overview of Risk Communication, see Balog-Way, McComas, and Besley 2020).

Although the AnthroShift is consistent with other perspectives that consider risk as a social pivot, it is unique in that it also assumes that social change in response to risk pivots are multi-directional. Society can shift to an orientation of social actors that prioritize environmental protections and the emergence of an “environmental state” (Frank et al. 2000; Buttel 2000; Mol and Buttel 2002). However, it can also move back to less environmentally sustainable practices (Fisher and Jorgenson 2019: 250).

As the world became accustomed to the risks associated with COVID-19, individuals resumed more normal levels of travel and social mixing even as the disease continued to spread. Consequently, the environmental co-benefits of the responses to the pandemic were short-lived and many states shifted back to pre-pandemic policies. This flip-flop in policies and behaviors provides a clear example of the multi-directionality of the AnthroShift. As a result, global greenhouse gas emissions quickly returned to their original upward trajectory with emissions in 2021 overshooting expectations (Liu et al. 2022). In their comment in *Nature Reviews Earth & Environment,* Liu and colleagues point out that the “rebounds are apparent in most sectors and big emitting nations.” The authors go on to note that, although the degree to which the pandemic affected emissions was “unprecedented, such crises and rebounds are not unique” (Liu et al. 2022, 1).

While greenhouse gas emissions resumed their climb, the planet continued to heat up. We witnessed numerous examples of the consequences of a changing climate in the form of extreme weather events around the globe in 2021, including hurricanes, droughts, floods, heatwaves, and wildfires. These consequences are documented in detail in the recently released Sixth Assessment Report from Working Group 2 of the Intergovernmental Panel on Climate Change (IPCC) on impacts, adaptation, and vulnerability (Intergovernmental Panel on Climate Change (IPCC) Working Group 2 2022). In the words of this report’s government-approved summary for policymakers: “Any further delay in concerted global action will miss the brief, rapidly closing window to secure a liveable future” (Intergovernmental Panel on Climate Change (IPCC) Working Group 2 2022, SPM-35).

## From COVID-19 to the Climate Crisis

Lessons regarding how the world responded to the pandemic provide important insights into what it will take to motivate the social responses needed to address the climate crisis (see also Manzanedo and Manning 2020). As previously noted, early responses to the pandemic had the unintended side effect of reducing greenhouse gas emissions. It is important to stress that these side effects were accidental; mitigating climate change was not directly connected to policy responses to COVID-19. Outside this example, there is extensive evidence that current efforts to address climate change are woefully inadequate from the state, market, and civil society sectors (see especially Intergovernmental Panel on Climate Change (IPCC) Working Group 3 2022; for a broad overview of the pathways and options for mitigation and adaptation, see also Jorgenson et al. 2019).

Governments have been working since 1992 to limit the effects of climate change and research has documented the role that transnational climate governance could play along with what has come to be known as polycentric approaches to governance systems in solving the climate crisis (For an overview, see Bulkeley et al. 2014; Cole 2015; A. J. Jordan et al. 2015; but see Fisher and Leifeld 2019). Even with numerous efforts to address the climate crisis at multiple levels of governance; however, policymaking has been relatively ineffective at bringing about the emissions reductions necessary to limit global warming below 1.5°C (A. Jordan et al. 2022; see also Intergovernmental Panel on Climate Change (IPCC) Working Group 3 2022). Although world leaders were expected to coordinate a global response to the climate crisis that met the challenges the world was facing at the delayed COP-26 climate negotiations in Glasgow in November 2021, the outcome was disappointing. Countries were unable to agree to the emissions reductions needed to respond to the dire warnings of Working Group 1 of the IPCC, which released its Sixth Assessment Report before the international negotiations began in 2021 (Intergovernmental Panel on Climate Change (IPCC) Working Group 1 2021). After 2 weeks of muddled negotiations, countries punted. They are now expected to meet in November 2022 at COP-27 in Egypt to finalize their plans for climate action that will limit global warming below the 1.5° threshold that scientists have determined will keep the most dire effects of climate change at bay (UNEP 2021a).

At the domestic level, even though it is abundantly clear that countries vary substantially regarding type of regime and institutional makeup (See especially Lachapelle and Paterson 2013; Lockwood et al. 2017; Ward et al. 2014), sufficient climate action for most countries continues to be out of reach (Intergovernmental Panel on Climate Change (IPCC) Working Group 3 2022). In the USA, for example, the country is expected to overshoot its original climate commitments set by President Obama at the Paris round of the climate negotiations in 2015. These targets were classified as “insufficient” by the independent scientific team at Climate Action Tracker to keep global warming below 3°C.^1^ The Biden Administration submitted an updated commitment prior to the COP-26 round of negotiations that gets closer to achieving the emissions reduction goals, but the country has yet to pass policies that would meet these commitments, let alone implement them.

Outside the USA, it is not much better. Although many other developed countries have filed Nationally Determined Contributions (NDCs) that indicate plans to reduce their emissions in line with the IPCC’s targets, the implementation of policies that achieve these intended goals are few and far between. As of late October 2021, the UN Emissions Gap Report noted that national climate pledges going into the COP-26 negotiations were still “well above the goals of the Paris climate agreement and would lead to catastrophic changes in the Earth’s climate” (UNEP 2021b). A more recent study documents how countries in the G20 have not followed through on their commitments to invest in emissions reductions during the COVID recovery (Nahm et al. 2022). Moreover, many of the countries that have committed to cut emissions within their borders continue to permit fossil fuel expansion with the intent of exporting these resources to other parts of the world (Energy Mix 2022). The lack of sufficient climate action was noted by UN Secretary General during the release of the Sixth Assessment Report from IPCC Working Group 3 on Mitigation in April 2022. In his words, the report documents “'a litany of broken climate promises' by governments and businesses.”^2^ The war in Ukraine has exacerbated this process as countries scramble to increase fossil fuel production outside Russia, rather than encouraging a faster shift to clean energy sources (see discussion in Ayala 2022).

At the same time that state responses have been insufficient, business efforts have been bi-polar. In contrast to the swift and effective global response to Ozone depletion, where a technological fix was discovered and companies encouraged governments to implement it (Benedick 1998; for an alternative perspective see Litfin 1994; see also Oelschlaeger 1979 for a critical assessment of the technological fix), the climate crisis has no silver bullet. Instead, companies representing non-carbon emitting energy sources and technologies compete with entrenched business interests that continue to support an economy fueled by fossil fuels. A growing literature documents how these vested economic and political interests have worked to maintain the status quo through the process of agency and regulatory capture (Freudenburg and Gramling 1994; Dillon et al. 2018; Hertel-Fernandez 2019; Mildenberger 2020), as well as by employing dark money that funds and disseminates climate misinformation that aims to confuse and distract from climate action (Jacques et al. 2008; Brulle 2014; Farrell 2016, 2019; Supran and Oreskes 2017).

Even as big oil companies have started to acknowledge the climate issue and propose plans to address climate change, their so-called “zero emissions” plans do not provide comprehensive strategies. Specifically, these plans intentionally omit reductions in what they call “scope 3 emissions” derived from the actual burning of the natural resources that they are extracting and selling. In other words, these companies have developed plans to reduce the emissions associated with the *extraction* of the fossil fuels, but do not consider the emissions from the *burning* of these fuels since they are sold to other companies and/or countries (for more detail, see Hertwich and Wood 2018).^3^

Given the lack of meaningful progress to reduce emissions from the state and market sectors over the past 30 years, there has been growing pressure from civil society, which includes a range of non-state actors, such as non-governmental organizations (NGOs), unions, social movements, as well as individual activists (Kuyper et al. 2018; Spencer et al. 2018; de Moor et al. 2020). Consequently, climate activism has become more common in recent years and has employed a range of tactics including climate strikes, protests, and boycotts that aim to raise awareness of the climate issue and motivate climate action.^4^ In September 2019, for example, an estimated 7.6 million people participated in the “Global Week for Future” actions around the world that were scheduled to coincide with the UN Climate Action Summit (Rosane 2019; see also Fisher and Nasrin 2021). To date, however, the effects of climate activism on economic and political actors’ behaviors has been relatively spotty (for a full discussion, see Fisher and Nasrin 2021). Even less is known about the actual climate effects of these efforts in terms of emissions (Fisher and Nasrin 2021). The research that does analyze how civic action affects carbon emissions and concentrations faces numerous “methodological challenges as the data on the activism, as well as on the connections between the activism and its effects on greenhouse gas emissions are both limited” (Fisher and Nasrin 2021: 6). In other words, although climate activism holds promise to pressure state and market actors to respond meaningfully to the climate crisis, much more research is needed to measure what sorts of climate activism are most effective at leading to reductions in greenhouse gas emissions and how.

## Getting to an AnthroShift

In summary, there is insurmountable evidence that current responses to the climate crisis from the state, market and civil society sectors will not save us from a warming world. At the same time, carbon concentrations in the atmosphere will continue to rise and the effects of climate change will be felt with more frequency (Intergovernmental Panel on Climate Change (IPCC) Working Group 2 2022). Given this current level of inaction, it is very likely that only an AnthroShift driven by a substantial risk pivot can put us on a more sustainable path. But what will it take to get there?

Even though the environmental side-effects of the responses to COVID-19 were unintentional, the way society responded to the COVID-19 pandemic provides some guidance. Risk only motivates an AnthroShift if it surpasses a threshold in terms of its duration and intensity. Moreover, without a sustained shock that has tangible consequences in terms of social cost to people and property, the subsequent social change will be ephemeral. In those cases, the reorientation of social actors will shift back towards the original configuration that maintains a business-as-usual trajectory. In the case of climate change, this trajectory is supported and encouraged by the many actors with vested interests that have consolidated access to resources and power through the fossil fuel-based economy (see e.g., Mildenberger 2020).

The change required to keep global warming below 1.5°C as outlined by the IPCC is substantial. It requires both halting the emissions of greenhouse gases along with the adoption of some “negative-emissions technologies” to remove the greenhouse gases already in the atmosphere (Rueda et al. 2021), which have yet to be proven to work at the scale necessary. This degree of social change is impossible without a radical transformation in the ways that the state, market, and civil society sectors interact. The most common examples of drastic social change in response to crisis are war and/or economic depression (e.g., Tilly 1985; Polanyi 2001; Halperin 2004; but see Foran and Goodwin 1993).

Barring a world war or widescale economic depression, both of which have been noted in the IPCC’s most recent report as possible consequences of climate change (see particularly Intergovernmental Panel on Climate Change (IPCC) Working Group 2 2022, chap. 12), the type of radical social change needed is most likely to be initiated by civil society. A global mass mobilization that employs either nonviolent or more confrontational tactics has the potential to motivate the type of social transformation needed. Nonviolent conflict has been found to be successful in bringing about such large-scale social transformations if a critical mass of 3.5% or more of the population participates in the activism (see e.g., Chenoweth and Stephan 2011). To date, however, beyond responses to repressive and autocratic rule, there are very few examples of sustained activism at this level of engagement. Accordingly, it is unrealistic to imagine that this percentage of the population of many countries would mobilize and engage in peaceful climate activism without some sort of large-scale risk pivot to motivate it.

It is possible that less peaceful forms of activism could lead to the kinds of radical social change that are needed to address the climate crisis. In fact, calls for such confrontational activism are growing (see particularly Malm 2021). It is worth noting that, even when activism starts out peaceful, disruptive tactics like roadblocks, sit-ins, and human barriers that are not violent run the risk of turning violent due to the ways that law enforcement responds; repression can easily lead to an escalation of violence on both sides (Bloom 2020; see also McAdam 1982).

A large-scale risk pivot can lead to peaceful and less-peaceful mass mobilizations at the levels needed to stimulate an AnthroShift. Only a global risk event (or numerous smaller events that are seen as threatening social and economic centers of power) will motivate the massive social change needed. In other words, without a risk pivot—be it driven by social or environmental change—an AnthroShift that is large enough to respond adequately to the climate crisis is improbable. At this point, it is impossible to predict if such a shock will come from ecological disaster, war, pandemic, or another unforeseen risk. What is certain, though, is that without such a shock that motivates an AnthroShift that reorients all the sectors of society to respond meaningfully, it is hard to envision the world achieving the levels of climate action needed.

## Data Availability

Not applicable

## Footnotes

1 https://climateactiontracker.org/countries/usa/ (Accessed 21 December 2021).

2 https://www.theguardian.com/environment/video/2022/apr/04/world-on-fast-track-to-climate-disaster-say-un-secretary-general-video (Accessed 7 April 2022).

3 https://www.spglobal.com/commodity-insights/en/market-insights/latest-news/oil/092021-oil-majors-pledge-net-zero-target-update-goals-to-cut-methane-carbon-intensity (Accessed 4 March 2022)

4 This list is not meant to be exhaustive.

## References

[CR1] Ayala C (2022) Former navy secretary: the addiction to fossil fuels empowers Putin. Text. TheHill. March 2, 2022. https://thehill.com/opinion/energy-environment/596336-former-navy-secretary-the-addiction-to-fossil-fuels-empowers-putin.

[CR2] Balog-Way D, McComas K, Besley J (2020). The evolving field of risk communication. Risk Analysis.

[CR3] Beck U (1997). Subpolitics: ecology and the disintegration of institutional power. Org Environ.

[CR4] Beck U (1999) World Risk Society. 1 edition. Malden, MA: Polity

[CR5] Beck U, Giddens A, Lash S (1994). Reflexive modernization: politics, tradition and aesthetics in the modern social order.

[CR6] Benedick RE (1998). *Ozone Diplomacy*.

[CR7] Bhat SA, Bashir O, Bilal M, Ishaq A, Din MU, Dar RK, Bhat RA, Sher F (2021). Impact of COVID-related lockdowns on environmental and climate change scenarios. Environ Res.

[CR8] Bloom J (2020). The dynamics of repression and insurgent practice in the Black liberation struggle. Am J Sociol.

[CR9] Brulle RJ (2014). Institutionalizing delay: foundation funding and the creation of U.S. climate change counter-movement organizations. Climatic Change.

[CR10] Bulkeley H, Andonova LB, Betsill MM, Compagnon D, Hale T, Hoffmann MJ, Newell P, Paterson M, Roger C, VanDeveer SD (2014). *Transnational Climate Change Governance*.

[CR11] Buttel FH (2000). World society, the nation-state, and environmental protection: comment on Frank, Hironaka, and Schofer. Am Sociolo Rev.

[CR12] Chenoweth E, Stephan MJ (2011). *Why civil resistance works: the strategic logic of nonviolent conflict*.

[CR13] Cole DH (2015). Advantages of a polycentric approach to climate change policy. Nat Climate Change.

[CR14] Curran D (2013). Risk society and the distribution of bads: theorizing class in the risk society. The British Journal of Sociology.

[CR15] Dillon L, Sellers C, Underhill V, Shapiro N, Ohayon JL, Sullivan M, Brown P, Harrison J, Wylie S (2018). The environmental protection agency in the early Trump administration: prelude to regulatory capture. Am J Public Health.

[CR16] Ekberg M (2007). The parameters of the risk society: a review and exploration. Curr Sociol.

[CR17] Elliott JR, Frickel S (2015). Urbanization as socioenvironmental succession: the case of hazardous industrial site accumulation. Am J Sociolo.

[CR18] Farrell J (2016). Corporate funding and ideological polarization about climate change. Proceed Nat Acad Sci.

[CR19] Farrell J (2019). The growth of climate change misinformation in US philanthropy: evidence from natural language processing. Environ Res Letters.

[CR20] Fisher DR, Jorgenson AK (2019). Ending the stalemate: toward a theory of anthro-shift. Sociolo Theory.

[CR21] Fisher DR, Leifeld P (2019) The polycentricity of climate policy blockage. Climatic Change, July. 10.1007/s10584-019-02481-y, 155, 4, 469, 487

[CR22] Fisher DR, Nasrin S (2021). Climate activism and its effects.

[CR23] Foran J, Goodwin J (1993). Revolutionary outcomes in Iran and Nicaragua: coalition fragmentation, war, and the limits of social transformation. Theory and Society.

[CR24] Frank DJ, Hironaka A, Schofer E (2000). The Nation-State and the Natural Environment over the Twentieth Century. Am Sociolo Rev.

[CR25] Freudenburg WR, Gramling R (1994). Bureaucratic slippage and failures of agency vigilance: the case of the environmental studies program. Soc Problems.

[CR26] Goering L (2020). Greta Thunberg says coronavirus shows world can act fast on crises. Global Citizen. March.

[CR27] Halperin S (2004). *War and social change in modern Europe: the great transformation revisited*.

[CR28] Hertel-Fernandez A (2019). State capture: how conservative activists, big businesses, and wealthy donors reshaped the American states -- and the nation.

[CR29] Hertwich EG, Wood R (2018). The growing importance of scope 3 greenhouse gas emissions from industry. Environ Res Letters.

[CR30] Hosseini SE (2020). An outlook on the global development of renewable and sustainable energy at the time of COVID-19. Energy Res Soc Sci.

[CR31] Hultman NE, Hassenzahl DM, Rayner S (2010). Climate risk. Ann Rev Environ Resources.

[CR32] Intergovernmental Panel on Climate Change (IPCC) Working Group 1 (2021). Climate Change 2021: The Physical Science Basis. Contribution of Working Group I to the Sixth Assessment Report of the Intergovernmental Panel on Climate Change.

[CR33] Intergovernmental Panel on Climate Change (IPCC) Working Group 2 (2022). Climate Change 2022: Impacts, Adaptation, and Vulnerability. Contribution of Working Group II to the Sixth Assessment Report of the Intergovernmental Panel on Climate Change.

[CR34] Intergovernmental Panel on Climate Change (IPCC) Working Group 3. 2022. Climate Change (2022) Mitigation of Climate Change. Contribution of Working Group III to the Sixth Assessment Report of the Intergovernmental Panel on Climate Change. Cambridge: Cambridge University Press. doi: 10.1017/9781009157926.

[CR35] Jacques PJ, Dunlap RE, Freeman M (2008). The organisation of denial: conservative think tanks and environmental scepticism. Environmental Politics.

[CR36] Jordan AJ, Huitema D, Hildén M, van Asselt H, Rayner TJ, Schoenefeld JJ, Tosun J, Forster J, Boasson EL (2015). Emergence of polycentric climate governance and its future prospects. Nat Climate Change.

[CR37] Jordan A, Lorenzoni I, Tosun J, Saus JE i, Geese L, Kenny J, Saad EL, Moore B, Schaub SG (2022). The political challenges of deep decarbonisation: towards a more integrated agenda. Climate Action.

[CR38] Jorgenson AK, Fiske S, Hubacek K, Li J, McGovern T, Rick T, Schor JB, Solecki W, York R, Zycherman A (2019). Social science perspectives on drivers of and responses to global climate change. WIREs Climate Change.

[CR39] Kuyper JW, Linnér B-O, Schroeder H (2018). Non-state actors in hybrid global climate governance: justice, legitimacy, and effectiveness in a post-Paris era. WIREs Climate Change.

[CR40] Lachapelle E, Paterson M (2013). Drivers of national climate policy. Climate Policy.

[CR41] Litfin K (1994). Ozone Discourses: Science and Politics in Global Environmental Cooperation.

[CR42] Lockwood M, Kuzemko C, Mitchell C, Hoggett R (2017). Historical institutionalism and the politics of sustainable energy transitions: a research agenda. Environment and Planning C: Politics and Space.

[CR43] Malm A (2021). How to blow up a pipeline: learning to fight in a world on fire.

[CR44] Manzanedo RD, Manning P (2020). COVID-19: lessons for the climate change emergency. Science of The Total Environment.

[CR45] McAdam D (1982). *Political process and the development of black insurgency, 1930-1970*.

[CR46] Mildenberger M (2020). *Carbon captured: how business and labor control climate politics*. American and Comparative Environmental Policy.

[CR47] Energy Mix (2022) Record Fossil Extraction from Canada, U.S., Norway Despite Fervent Climate Pledges. The Energy Mix (blog). February 2, 2022. https://www.theenergymix.com/2022/02/02/record-fossil-extraction-from-canada-u-s-norway-despite-fervent-climate-pledges.

[CR48] Mol APJ, Buttel FH (2002) The environmental state under pressure: an introduction. In *The Environmental State Under Pressure*, edited by P.J. Mol Arthur and H. Buttel Frederick, 10:1–11. Research in Social Problems and Public Policy. Emerald Group Publishing Limited. 10.1016/S0196-1152(02)80003-5.

[CR49] Joost de M, De Vydt M, Uba K, Wahlström M (2020) New kids on the block: taking stock of the recent cycle of climate activism. Social Movement Studies, October, 1–7. 10.1080/14742837.2020.1836617.

[CR50] Nahm JM, Miller SM, Urpelainen J (2022). G20’s US$14-Trillion Economic Stimulus Reneges on Emissions Pledges. Nature.

[CR51] Oelschlaeger M (1979). The myth of the technological fix. Southwestern J Philosophy.

[CR52] Polanyi K (2001). The great transformation: the political and economic origins of our time.

[CR53] Rosa E, McCright A, Renn O (2015) The Risk Society Revisited: Social Theory and Risk Governance. Reprint edition. Philadelphia, Pa: Temple University Press

[CR54] Rosane O (2019) 7.6 million join week of global climate strikes. EcoWatch. September 30, 2019. https://www.ecowatch.com/global-climate-strikes-week-2640790405.html.

[CR55] Rueda O, Mogollón JM, Tukker A, Scherer L (2021) Negative-emissions technology portfolios to meet the 1.5 °C target. *Global Environmental Change* 67 (March): 102238. 10.1016/j.gloenvcha.2021.102238

[CR56] Sovacool BK, Del Rio DF, Griffiths S (2020). Contextualizing the Covid-19 pandemic for a carbon-constrained world: insights for sustainability transitions, energy justice, and research methodology. Energy Res Soc Sci.

[CR57] Spencer T, Colombier M, Sartor O, Garg A, Tiwari V, Burton J, Caetano T, Green F, Teng F, Wiseman J (2018). The 1.5°C Target and Coal Sector Transition: At the Limits of Societal Feasibility. Climate Policy.

[CR58] Stallings RA (1990). Media discourse and the social construction of risk. Social Problems.

[CR59] Su, Fenzhen, Dongjie Fu, Fengqin Yan, Han Xiao, Tingting Pan, Yang Xiao, Lu Kang, et al. 2021. Rapid greening response of China’s 2020 spring vegetation to COVID-19 restrictions: implications for climate change. Science Advances 7 (35): eabe8044. 10.1126/sciadv.abe8044. 10.1126/sciadv.abe8044 PMC8386938 34433554

[CR60] Supran, Geoffrey, and Naomi Oreskes. 2017. Assessing ExxonMobil’s climate change communications (1977–2014).” Environ Res Letters 12 (8): 084019. 10.1088/1748-9326/aa815f.

[CR61] Tilly C, Evans PB, Rueschemeyer D, Skocpol T (1985). War making and state making as organized crime. *Bringing the State Back In*.

[CR62] UNEP (2021a) COP26 ends with agreement but falls short on climate action. UNEP News and Stories. November 15:2021 http://www.unep.org/news-and-stories/story/cop26-ends-agreement-falls-short-climate-action

[CR63] UNEP (2021b) Emissions gap report 2021. UNEP - UN Environment Programme. October 25, 2021. http://www.unep.org/resources/emissions-gap-report-2021.

[CR64] Venter ZS, Aunan K, Chowdhury S, Lelieveld J (2020). COVID-19 Lockdowns Cause Global Air Pollution Declines. Proceed Nat Acad Sci.

[CR65] Ward H, Cao X, Mukherjee B (2014). State capacity and the environmental investment gap in authoritarian states. Comparative Political Studies.

[CR66] Zhu L, Deng Z, Davis SJ, Giron C, Ciais P (2022) Monitoring global carbon emissions in 2021. *Nature Reviews Earth & Environment*, March, 1–3. 10.1038/s43017-022-00285-w, Monitoring global carbon emissions in 2021 10.1038/s43017-022-00285-w PMC8935618 35340723

